# Ambient floor vibration sensing advances the accessibility of functional gait assessments for children with muscular dystrophies

**DOI:** 10.1038/s41598-024-60034-5

**Published:** 2024-05-11

**Authors:** Yiwen Dong, Megan Iammarino, Jingxiao Liu, Jesse Codling, Jonathon Fagert, Mostafa Mirshekari, Linda Lowes, Pei Zhang, Hae Young Noh

**Affiliations:** 1 Stanford University, Stanford, USA; 2 Nationwide Children’s Hospital, Columbus, USA; 3 University of Michigan, Ann Arbor, USA; 4 Baldwin Wallace University, Berea, USA

**Keywords:** Outcomes research, Paediatric research, Biomedical engineering, Civil engineering, Electrical and electronic engineering

## Abstract

Muscular dystrophies (MD) are a group of genetic neuromuscular disorders that cause progressive weakness and loss of muscles over time, influencing 1 in 3500–5000 children worldwide. New and exciting treatment options have led to a critical need for a clinical post-marketing surveillance tool to confirm the efficacy and safety of these treatments after individuals receive them in a commercial setting. For MDs, functional gait assessment is a common approach to evaluate the efficacy of the treatments because muscle weakness is reflected in individuals’ walking patterns. However, there is little incentive for the family to continue to travel for such assessments due to the lack of access to specialty centers. While various existing sensing devices, such as cameras, force plates, and wearables can assess gait at home, they are limited by privacy concerns, area of coverage, and discomfort in carrying devices, which is not practical for long-term, continuous monitoring in daily settings. In this study, we introduce a novel functional gait assessment system using ambient floor vibrations, which is non-invasive and scalable, requiring only low-cost and sparsely deployed geophone sensors attached to the floor surface, suitable for in-home usage. Our system captures floor vibrations generated by footsteps from patients while they walk around and analyzes such vibrations to extract essential gait health information. To enhance interpretability and reliability under various sensing scenarios, we translate the signal patterns of floor vibration to pathological gait patterns related to MD, and develop a hierarchical learning algorithm that aggregates insights from individual footsteps to estimate a person’s overall gait performance. When evaluated through real-world experiments with 36 subjects (including 15 patients with MD), our floor vibration sensing system achieves a 94.8% accuracy in predicting functional gait stages for patients with MD. Our approach enables accurate, accessible, and scalable functional gait assessment, bringing MD progressive tracking into real life.

## Introduction

Muscular dystrophies (MD) are a group of neuromuscular disorders that commonly present with muscle weakness and atrophy and result in motor and gait impairments of varying degrees. MDs are present in 1 in 3500–5000 children worldwide, depending on the type^1–3^. While there are no cures available for the spectrum of MDs, treatments such as corticosteroid therapy and proactive cardiac and respiratory intervention have been evidenced to delay the progression of the disease and can extend a child’s lifespan^4,5^. In recent years, the therapeutic landscape has been quickly evolving with the promise of various therapeutic modalities making their way through the clinical trial pipeline. However, the cycle of therapeutic development can be long, and long-term effectiveness is difficult to assess after conditional approval of their usage; long-term monitoring is critical to expand the accessibility of these treatments to all patients. For example, with the recent accelerated approval of Elevidys, a one-time gene replacement therapy for Duchenne muscular dystrophy (DMD) that has been shown to improve gross motor skill, there is an urgent unmet need for a way to collect functional gait outcome data on children who have received this gene therapy commercially. As Elevidys is a one-time infusion, there is little incentive for the family to continue to come for follow-up visits^6,7^. Additionally, there is a lack of access to specialty centers with experts who are trained to administer disease-specific assessments, limiting the ability to track the impact of treatment over time, especially for low-income families. To this end, a monitoring system that passively tracks the progression of MD at children’s homes to assess their functional gait through the observation of gait symptoms can accelerate the post-approval process, closing the cycle of treatment development.

Previous studies have developed both clinical assessments and sensing technologies to monitor gait health in patients with MD. Existing clinical practice in tracking the progression of MD is by measuring the patient’s functional abilities, including walking/running speed or ability to do common activities such as climbing stairs^8,9^. However, these assessments require in-person appointments at specialized clinics (around 150 in the U.S.), which may not be accessible to low-income families or those who live in rural areas. Therefore, a system that can monitor patients in their homes is needed for more frequent and continuous monitoring of either MD progression and/or treatment effect. To achieve this, several existing sensing technologies are available, including force/pressure mat^10^, wearable devices^11^, and camera-based systems^12^; however, they have either limited spatial coverage, are unable to run for a long period, or have limited kinetic information. For example, force/pressure sensors require dense deployment due to their restricted sensing range; wearables require patients to carry devices all the time, which is often challenging for children; cameras require clear lines of sight and lack kinetic information on how the forces act and transmit through the body. They also raise privacy concerns when installed at home. These limitations make such technologies less practical and inadequate for continuous monitoring in daily life.

In this study, we introduce a novel framework based on sensing ambient floor vibrations to monitor gait health in patients with MD. The physical mechanism behind this approach is that as a person walks and steps on the floor, each footstep exerts a force at the floor surface that generates vibration waves. By capturing and analyzing these waves through vibration sensors mounted on the floor, the characteristics of a person’s gait can be inferred and various gait symptoms related to MD can be identified, as discussed in a previous study^13^. For example, waddling gait is commonly observed among children with MD, which is characterized by the sway of the body from side to side and hip drops with each step^14,15^. This leads to slower, unstable walking that is reflected in floor vibration through larger intervals between the vibration impulses and larger variances among a series of continuous footstep signals. The main advantages of our approach include high scalability, low cost/maintenance, contactless data collection, and wide coverage (up to 20 m distance as evaluated in previous work using the same sensor type^16^), leading to more accessible functional gait assessments in patients’ homes. Furthermore, it is perceived as more privacy-friendly, and thus suitable for in-home usage.

The main research challenges to relate the signal patterns of floor vibration to pathological gait patterns in patients with MD, mainly lie in two aspects: (1) interpretability and (2) complex influencing factors in gait. First, unlike camera and force plate measurements, vibration signals do not provide direct observations of the motion and force in a person’s gait. Therefore, it is necessary to analyze and interpret the vibration signals in terms of gait characteristics to produce clinical insights. Secondly, one unique challenge in long-term continuous gait monitoring is the variability in walking patterns due to a complex mixture of reasons in daily life. For example, gait patterns are not only influenced by neuromuscular disorders, but also by a person’s energy level, emotional status, and environmental disturbances^17–19^. As a result, it is difficult to incorporate all these factors when assessing an individual’s gait, which could lead to erroneous estimations of functional outcomes for clinical trials.

To overcome these challenges, we develop our framework to (1) improve the interpretability of vibration signals by characterizing and extracting gait symptoms related to MD from floor vibration data (discussed in Sect. 2.2), and (2) enhance its robustness to the complex influencing factors in gait by formalizing and integrating multiple levels of gait and biometric features from an individual through a hierarchical deep-learning model to make a collective decision (discussed in Sect. 2.3). Specifically, we first extract the gait features that explicitly inform symptoms related to MD, including cadence, symmetry, and initial contact types (see Sect. 2.2). Then, these explicit gait symptom-based features are combined with the implicit signal-based features (e.g., dominant frequencies, power spectral density, energy variability) to make a decision collaboratively. To reduce fluctuations in our prediction of gait health due to the complex influencing factors, we leverage the power of data aggregation and develop a model with a hierarchical structure that aggregates the latent representations of all footstep signals from a person. This allows the model to capture the overall gait pattern of a person instead of focusing on one single footstep. Specifically, our model first aggregates features from individual footsteps to a footstep trace (which consists of a series of consecutive footsteps), then combines the latent features from multiple footstep traces to produce a representative walking pattern of a person. After each level of aggregation, new features extracted from the current hierarchy are concatenated with the aggregated features from the previous hierarchy, which captures the variability and inter-dependencies between individual footsteps or traces. This provides additional information on gait balance and symmetry, which are important aspects of gait health.

We evaluate the framework through three rounds of walking experiments with 36 human subjects. The first round is a lab-based walking study to test the feasibility of floor vibration in capturing MD gait symptoms; The second and third rounds are conducted at a children’s hospital, to evaluate the performance of our sensing system with MD specialists and patients. Among all human subjects, 15 of which are children with various types of MD, including Duchenne, Becker, and Limb-Girdle MD. The rest of the subjects are healthy participants, including siblings of these children as well as adult volunteers. Our floor vibration sensing system achieved an average accuracy of 94.8% in tracking progression stages defined based on the 100-m run time measured during standard functional assessments in the hospital^20^, which is one of the most important references to understand the functional ability of an individual with MD.

## Results

### Evaluation of the ambient floor vibration sensing method

**Figure 1:**
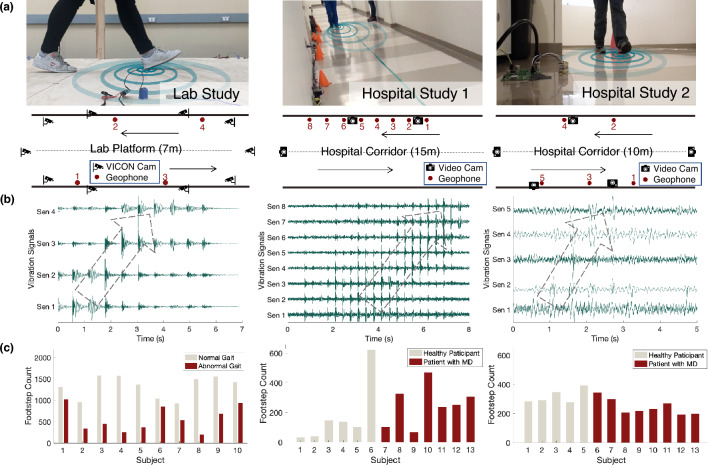
An overview of the floor vibration sensing evaluation set-ups, the recorded vibration data samples, and the participant cohorts, for the lab and hospital studies. (**a**) During the experiments, vibration sensors were placed on the floor near the edge of the walkways (represented by red dots). VICON Motion Capture System (for the lab study) and video cameras (for the hospital studies) were aimed at the subjects to capture the ground truth of gait characteristics. (**b**) Representative examples of the recorded vibration data from each of the setups show multiple series of footstep impulses resulting from a person’s gait. As the person walks from left to right, the peak amplitudes of the footstep impulses observed from sensor 1 to sensor *k* also shift from left to right over time (represented by the gray dashed arrows). (**c**) Summary of the participant cohort (N = 36 participants, N = 24416 footsteps) recorded in this study. Bars depict a subject-level breakdown of the number of footsteps from normal vs. abnormal gait or gait from patients with MD.

In order to evaluate the performance of the floor vibration sensing system in laboratory and real-life settings, we recruited human subjects ($N=36$) and conducted experiments at a university lab and a children’s hospital with healthy human subjects ($N=21$) and children with MD ($N=15$). The experiments consist of two phases - Phase 1 is a pilot study to test the feasibility of ambient floor vibration sensing in capturing gait characteristics in lab settings (Fig. 1 left column); Phase 2 has two hospital studies to evaluate our sensing method in real life (Fig. 1 middle and right columns).

The phase 1 lab study aims to explore the feasibility of using floor vibration signals in estimating gait parameters and detecting abnormal gait characteristics related to MD. We measured the normal gait and simulated abnormal gait characteristics related to MD from adult healthy participants ($N=10$) in a laboratory setting at the university. During the experiment, we installed four geophone sensors at the edge of a 7-meter-long walking platform, spaced 2 m apart (Fig. 1a), to capture the floor vibration induced by human gait. The sensor placement was designed to optimize the area of coverage and signal quality, as well as to provide redundancy in data collection and analysis. We also recorded the gait kinematics through the VICON Motion Capture system with 10 infrared cameras as the ground truth^21^, which has a less than 2mm measurement error as reported by existing studies^22,23^. The kinematics data were processed through manual labeling of gait cycles (i.e., foot strike and foot off time) and Plug-In Gait processing pipelines to compute the gait parameters^24^. Each participant walked back and forth on the platform for 20 trials, first with their natural gait on their own shoes, and then walking with simulated abnormal gait patterns that are commonly observed in patients with MD, including slow walking, asymmetrical walking, and toe/midfoot strike for 10–15 trials. The simulated walking trials were instructed by medical experts from gait clinics to ensure they were realistic. By analyzing the floor vibration data, our approach has a root-mean-square error (RMSE) of 0.05 s ($\sim 5$%) in estimating the step time and 0.15 m ($\sim 15$%) in estimating the step length. The error rates in estimating these spatiotemporal parameters are satisfactory and comparable to the state-of-the-art wearable devices^11^, which will be discussed further in Sect. 3. The accuracy for gait abnormality detection is up to 93% after testing on a data-driven model trained through frequency-domain features from the vibration signals.

We conducted the phase 2 study at the children’s hospital and evaluated our system in a real-life setting along a hospital corridor. The participants include patients with MD ($N=15$, 11 male and 4 female, age = 7–15 years, with $N_{DMD}=11$, $N_{BMD}=2$, $N_{LGMD}=2,$ for Duchenne, Becker, and Limb-Girdle MD, respectively) and other healthy subjects ($N=11$) such as the patient’s healthy siblings and the staff at the hospital. The healthy adults are considered to approximate teenagers who typically have similar walking patterns as the adults. To explore different sensor layouts, we deployed the system in two rounds, one with eight sensors placed in a straight line at one side of the corridor with 1 m apart, and another with five sensors mounted at both sides of the corridor, spaced with 2-m offsets (Fig. 1a). The sensor placement was significantly denser than required to provide redundancy in data collection and analysis. In both settings, 3–5 cameras were mounted around the sensing area, aiming at the lower body of the participants to collect the ground truth of their gait patterns. Each participant walked along the corridor back and forth for 5–10 rounds using their natural gait with their choice of footwear. For young children who have a high risk of falling, their parents walked along with them to provide timely assistance. The vibration signals generated by the parent were separated from the child through a recursive blind source separation algorithm developed in prior work^25^, in which the parent-only walking data were collected first as a template, and was then separated from the combined signal when walking with the child.

To evaluate the performance of the floor vibration sensing system, a unified scale for MD progression was defined based on the 100-m run time measured during standard functional assessments in the hospital^20^, which is one of the most important references to understand the functional ability of a patient. The scale divides the normative 100 m run time % into 8 functional stages (from Stage 0 to 7) based on their distinctive clinical interpretations, which is presented in Sect. 4.2. Since patients in the last two stages typically require walking assistance, our floor vibration sensing system estimates the functional stages from 0 to 5 for each subject. The distribution of stages among the subjects is—five participants in Stage 1, four participants in Stage 2, one participant in Stage 3, three participants in Stage 4, and two participants in Stage 5.

Our system achieves up to 94.8% test accuracy in classifying the functional stages using the data from 3 sensors. Using only one sensor (with the best data quality) still leads to more than 90% accuracy for MD functional stage prediction, which means that the number of sensors can be reduced significantly in practice. The second sensor layout setting has a slightly higher accuracy ($\sim 3\%$) than the first one because it captures more accurate spatial information of the gait when placing sensors at both sides.

### Characterization of gait patterns using floor vibration data

**Figure 2:**
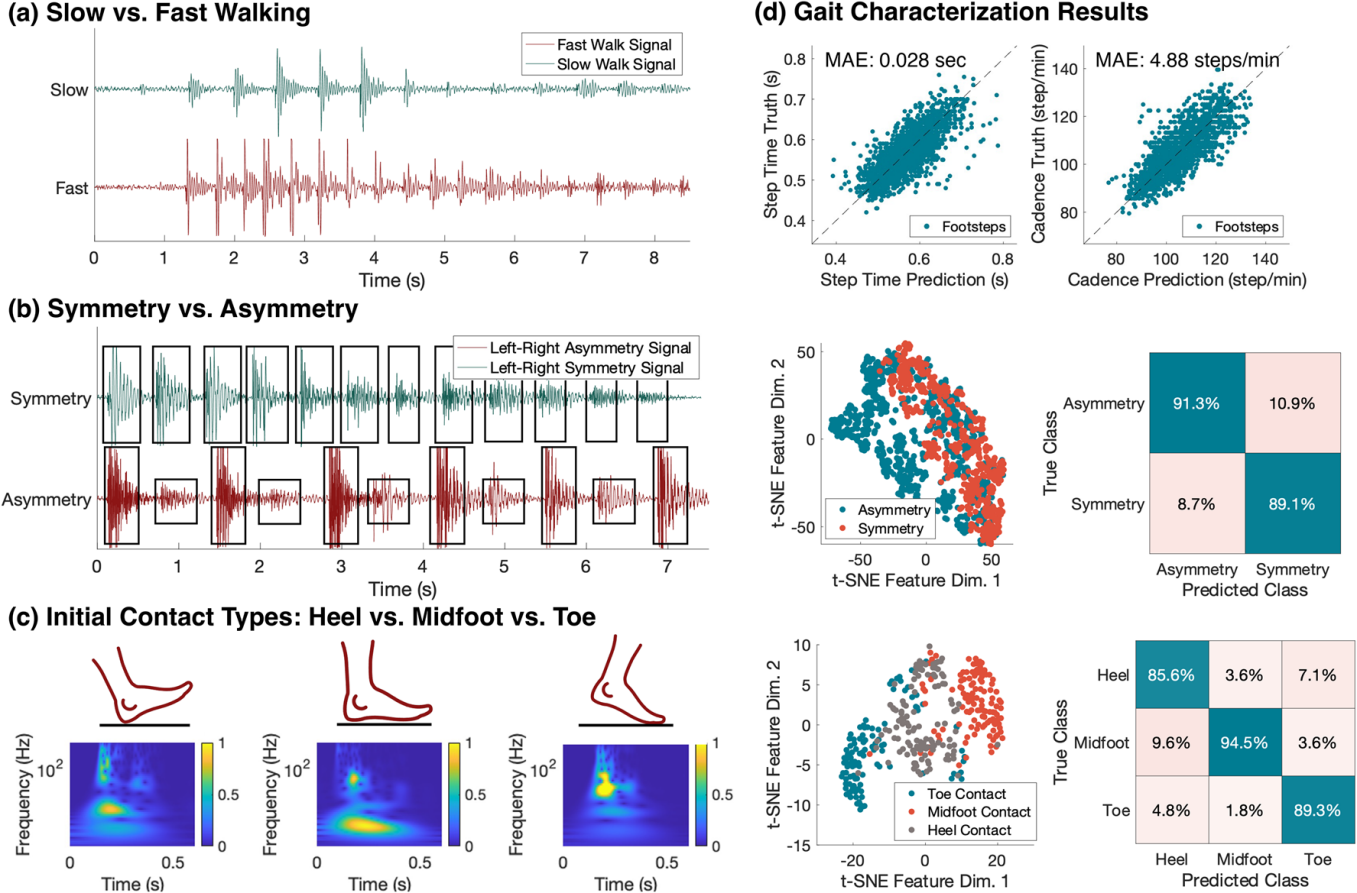
Gait characterization using floor vibration data and results for extracting MD symptom-based features. (**a**) Comparison between slow and fast walking. The fast walking signal has more peaks within a given amount of time than the slow walking signal. (**b**) Comparison between a healthy participant’s normal walking (symmetry) and when walking with an ankle brace on one leg (asymmetry). The asymmetry signal shows a significant energy difference between adjacent footstep impulses (as observed in the boxed footstep impulses). (**c**) Comparison between typical footsteps induced by heel, midfoot, and toe initial contacts. The difference in the signal frequency distribution over time observed in vibration time series demonstrates distinct contact patterns due to their unique contact surfaces and forces. (**d**) Summary of the gait characterization results. The first row shows the consistency between the predicted step time and cadence compared to the ground truth (N = 2003 footsteps), with 0.028 s and 4.88 step/min mean absolute errors (MAE) in step time and cadence estimation, respectively. The second and third rows show the t-SNE plots (left) and the confusion matrices of classification (right) obtained using the frequency spectrum of the vibration signals (N = 1647 footsteps). Note that the asymmetry plot overlaps the symmetry by around half of the samples because the vibration generated by the leg without the ankle brace matches the vibration from normal walking. Confusion matrices show the precision values (in %) computed based on the output of symmetry and initial contact estimation algorithms and the ground truth labeled by human experts. Note that the high precision values along the diagonal lines of both confusion matrices indicate a high level of agreement between the predictions and the ground truths.

**Figure 3:**
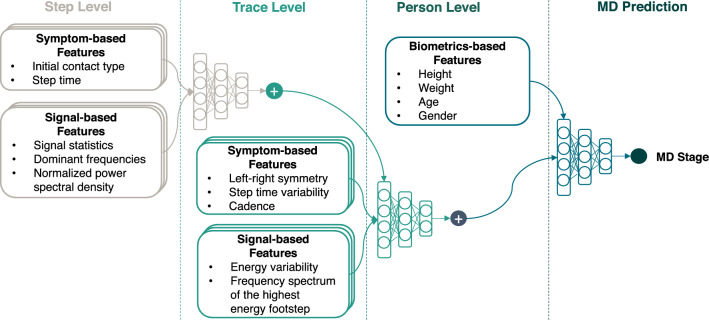
Block diagram of the hierarchical feature aggregation model. The step level includes symptom-based and signal-based features extracted from individual footsteps, which are highly variable but contain rich gait information. The multiple feature layers in the diagram indicate the parallel operation among multiple sensors. The combination of all step-level features serves as the input to the trace-level aggregation neural network. The trace level includes features from step-level aggregation, symptom-based and signal-based features describing the characteristics of a trace. Similarly, the multiple layers in the diagram indicate the parallel operation among multiple sensors. A multi-layer neural network aggregates the step-level features and generates an embedding for each trace. This embedding is then concatenated with the trace-level features to form the input to the person-level aggregation neural network. The person level includes features from the trace-level aggregation and additional biometrics information which affects a person’s gait pattern. Another multi-layer neural network aggregates the trace-level features and generates an embedding for each person. Similarly, this embedding is then concatenated with the biometric-based features to form the input to the final prediction neural network. The final prediction is made through a multi-layer neural network that aggregates each individual’s step-level, trace-level, and person-level to produce the final prediction of the MD functional stages.

To translate the floor vibration signals into gait health insights, we develop gait symptom-based features based on the vibration signals that explicitly inform gait parameters and health indicators (e.g., step time, cadence, initial contact type), as summarized in Fig. 2. The method to extract these symptoms is discussed in Sect. 4.4. In addition, we further extract signal-based features (e.g., frequency spectrum, signal energy) that represent the implicit patterns that are helpful yet undefined in existing gait assessments. By combining both explicit and implicit features, we advance our data-driven model using neural networks, which establish the complex nonlinear relationships between the features and the gait (Fig. 3). The data-driven model predicts the functional stages of individuals with MD by encoding the information from all the features. Evaluation results show that the combination of features produces a $2.1\times$ to $5.9\times$ error reduction compared to models with symptom-based or signal-based features only. The combined feature has a 94.8% accuracy, while the model with only signal-based or only symptom-based features has an average of 64.4% and 83.7% accuracy, respectively.

The extracted gait symptom-based features include step time, cadence, step time variability, left–right symmetry, and initial contact type. We estimated the step time as the duration between the foot strike impulse from one leg and the following impulse resulting from the contralateral leg and calculated the cadence by counting the number of foot strike-induced impulses within every minute (Fig. 2a). Both features (step time and cadence) are used to indicate how fast a person walks as numerous previous studies suggest that one significant sign of early-stage MD is the decrease in cadence and increase in step time^26^. We also estimated the step time variability through the standard deviation of the step time within each walking trace. This metric is found to be correlated to the balance of the gait^27,28^. In addition, severe left–right asymmetry has been previously observed in patients with MD in previous studies^29^, which was helpful in determining the need for assistive equipment for the patient in daily walking. In this study, we extracted the symmetry metric by computing the cosine distance between the frequency spectrum of the left and right foot, which compares the relative frequency distribution between these feet normalized by their corresponding foot strike energy (Fig. 2b). Finally, we predicted the probability of each initial contact type (toe vs. flatfoot vs. heel contact) by training on the data collected in the laboratory setting (Fig. 2c). Clinical studies have found that toe-walking, calf hypertrophy, and using the Gower’s maneuver to get up off of the floor are common early signs of MD. During gait assessments, the observation of abnormal initial contact (e.g., flatfoot and toe contact) with a wider base of support is commonly considered as a sign of MD progression^30^. When compared with the ground truth, the extracted symptom-based features demonstrated promising results of 0.028 s and 4.88 step/min mean absolute errors (MAE) for step time and cadence estimation, respectively, and 90.2% and 89.8% average precision in detecting asymmetry and toe/midfoot initial contacts, respectively (Fig. 2d).

The signal-based features include basic signal statistics, the dominant frequency at foot strike and foot off, power spectral density (frequency spectrum), and energy variability. The basic signal statistics include the mean, standard deviation, skewness, and kurtosis of the time-domain vibration signals from each footstep. These statistics provide an overview of signal patterns from various aspects, including intensity, variability, distribution patterns, and the proportion of extreme values. Then, we detected the dominant frequency at foot strike and foot off to gain insights into the ground reaction forces during the contact. As discussed in an existing study using floor vibration sensing^31^, a higher dominant frequency at the foot strike corresponds to frictional forces and a smaller contact area while the lower frequency indicates vertical forces and a larger contact area. The dominant frequency at toe push-off also indicates the power of the ankle plantarflexion (extension). The power spectral density is a spectrum of the signal’s power content versus frequency. It describes the power distribution over frequency normalized by the spectral resolution employed to digitize the signal, which has been shown to be representative in describing the implicit gait-related features encoded in various frequency components^13^. In addition, we computed the energy variability among a series of footsteps to describe the overall variability of a person’s footstep forces while walking because a stronger footstep force typically leads to a larger signal energy based on the elastic theory. Through evaluation, we found that signal-based features provide rich gait information on top of the symptom-based features, boosting the overall accuracy from 83.7 to 94.8%.

### Modeling hierarchical gait features for MD functional stage prediction

We developed and evaluated our hierarchical feature aggregation model to overcome the gait variability challenge induced by a complex mixture of physical and psychological influencing factors in daily life. We observed that a person’s gait pattern is hierarchical in nature—there are features describing individual footsteps (e.g., step time), features characterizing a trace of continuous footsteps (e.g., step variability), and factors that influence the overall pattern of a person’s gait (e.g., leg length, weight). Therefore, we incorporated this information by designing a hierarchical neural network that first integrates features from individual footsteps to a footstep trace, then combines the latent features from multiple footstep traces and a person’s unique biometrics to produce a representative feature set for that person’s gait. The benefit of the hierarchical architecture is to prompt the model to overcome the sampling bias among the participants by learning MD stages first through their gait characteristics and then through their biometrics. Given that there are unbalanced samples among various age groups, gender, body configurations, MD types, and so on, directly learning from all the features together may create artificial bias when estimating model parameters for MD stage assessments (e.g., the age may be over-emphasized because children with younger age tend to be at earlier stages of MD). By leveraging the domain knowledge of feature hierarchy and emphasizing the gait characteristics before considering the biometrics, we enable the hierarchical model to mitigate the effect of sampling bias by having the model learn from the gait features first. This makes the model more generalizable to other populations including previously unrecorded people.

Specifically, we categorized the symptom-based and signal-based features into two levels of hierarchy, including step-level and trace-level features, and then combined these with a person’s biometrics to predict the functional stage of MD (Fig. 3). The step-level features were extracted from individual footsteps, including the step time and initial contact type (symptom-based), as well as the basic signal statistics, dominant frequencies, and power spectral density (signal-based). The trace-level features were also computed based on a continuous series of footsteps, including the left–right symmetry, step time variability, cadence (symptom-based), signal energy variability among all footsteps, and the power spectral density of the most representative footstep (i.e., has the highest cumulative energy without clipping) of the entire trace (signal-based). The person-level features were obtained from medical records, mainly consisting of biometrics such as height, weight, age, and gender.

**Figure 4:**
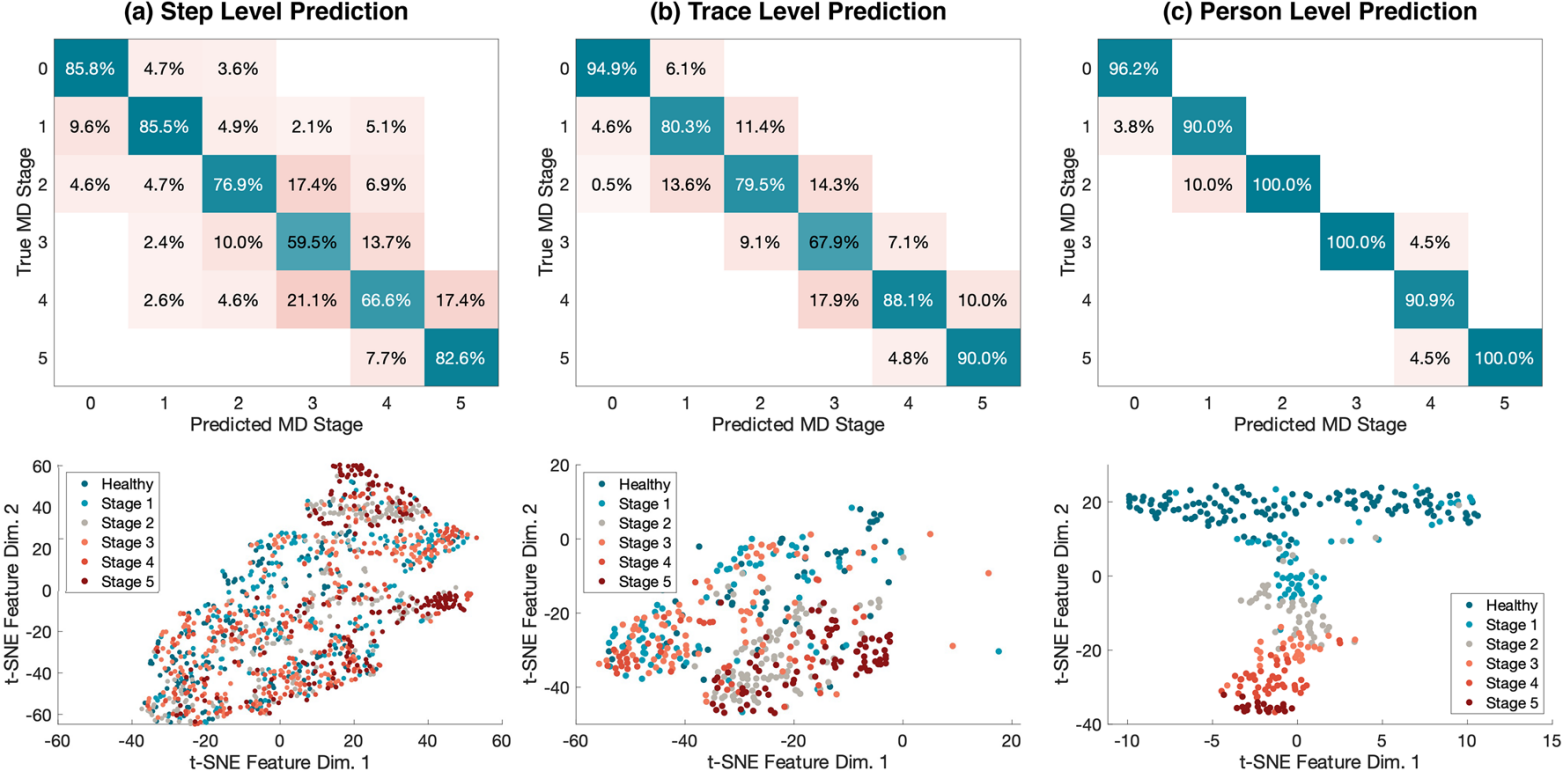
Classification accuracy of the hierarchical model and visualization of feature embeddings from step, trace, and person levels. The floor vibration sensing system makes predictions on the functional stages from 0 to 5 for each subject, and the results are compared with the ground truth. (**a**) The step level prediction only has an accuracy of 73.5% as the footstep has large variability. This can be observed from samples in the t-SNE plot where footsteps from different classes overlap with each other (N = 1425 footsteps). (**b**) The trace level prediction has slightly better performance than the step level with an 84.2% accuracy. This is because the features after the step-level aggregation reduce the variability, resulting in better separation between trace samples from various classes (N = 395 traces). (**c**) The person-level prediction shows the final results in MD stage prediction, which has a 94.8% accuracy. The t-SNE plot shows a distinct pattern from function stages 0 to 5 by combining trace-level and person-level features (N = 395 traces), which validates the efficacy of our model in predicting MD functional stages based on patients’ gait patterns.

At each level of the hierarchy, we utilized a multi-layer neural network to learn the embeddings of the features through training and testing on separated datasets. The training dataset consists of 80% of the randomly sampled footsteps, and the test dataset consists of the remaining 20%. The detailed neural network architecture and training procedure are introduced in Sect. 4.5. The confusion matrix and feature plots in Fig. 4 summarize the precision of footsteps/traces belonging to various functional stages under cross-validation on the test dataset, where the diagonal boxes show the percentage of correct predictions among all predicted values. We show that the hierarchical feature aggregation model is very effective in reducing false positives and mitigating the influence of the confounding factors—It increases the MD functional stage prediction accuracy from 73.5 (step level) to 84.2% (trace level), and finally to 94.8% at the person-level prediction ($\sim 5\times$ error reduction).

## Discussion

### Advantages of ambient floor vibration sensing for functional gait assessments

The ambient floor vibration sensing approach meets the requirements for functional gait assessment in patient’s home settings, providing accessible and accurate monitoring of gait health for individuals with MD. The floor vibration sensing system utilizes low-cost and portable geophone sensors attached to the floor surface that passively capture the gait-induced vibrations as a person walks by. This approach is contactless, non-interruptive, and unobtrusive, allowing long-term and continuous gait health monitoring in daily life. Our evaluation demonstrates that this sensing system can accurately estimate various gait parameters, infer gait symptoms, and produce quantitative and interpretable predictions on progressive stages of MD. The system was evaluated with a diverse group of subjects in terms of age, gender, and MD types, showing its potential to be extended to other diseases that involve gait disorders.

The floor vibration sensing approach has comparable error rates as the state-of-the-art sensing technologies while being more suitable for continuous and long-term gait monitoring at home. Previous studies have explored video cameras, force plate/pressure mats, wearable devices, and radio-frequency-based devices. These systems typically have around 0.01–0.1 s and 1–20 cm of error in temporal and spatial parameter estimation^10,11,23^. For comparison, the floor vibration sensing approach has an average of 0.05 s and 25 cm error for temporal and spatial parameter estimation, respectively. While the vibration sensing approach has limited spatial performance due to the interference of the floor structures, it has satisfactory accuracy in extracting temporal information and significant operational benefits, including having a larger area of coverage (up to 20 m), not carrying devices, being contactless and more privacy friendly. Therefore, this approach is suitable for in-home functional gait assessments that require acceptable accuracy while being more convenient and can operate in a longer term. To this end, it can advance the accessibility of functional gait assessment for low-income families or families living in areas where specialized clinics are unavailable. This will accelerate the treatment development cycle, increasing the enrollment rate while reducing the cost of post-approval studies.

### Analysis of feature trends with MD functional stages

**Figure 5:**
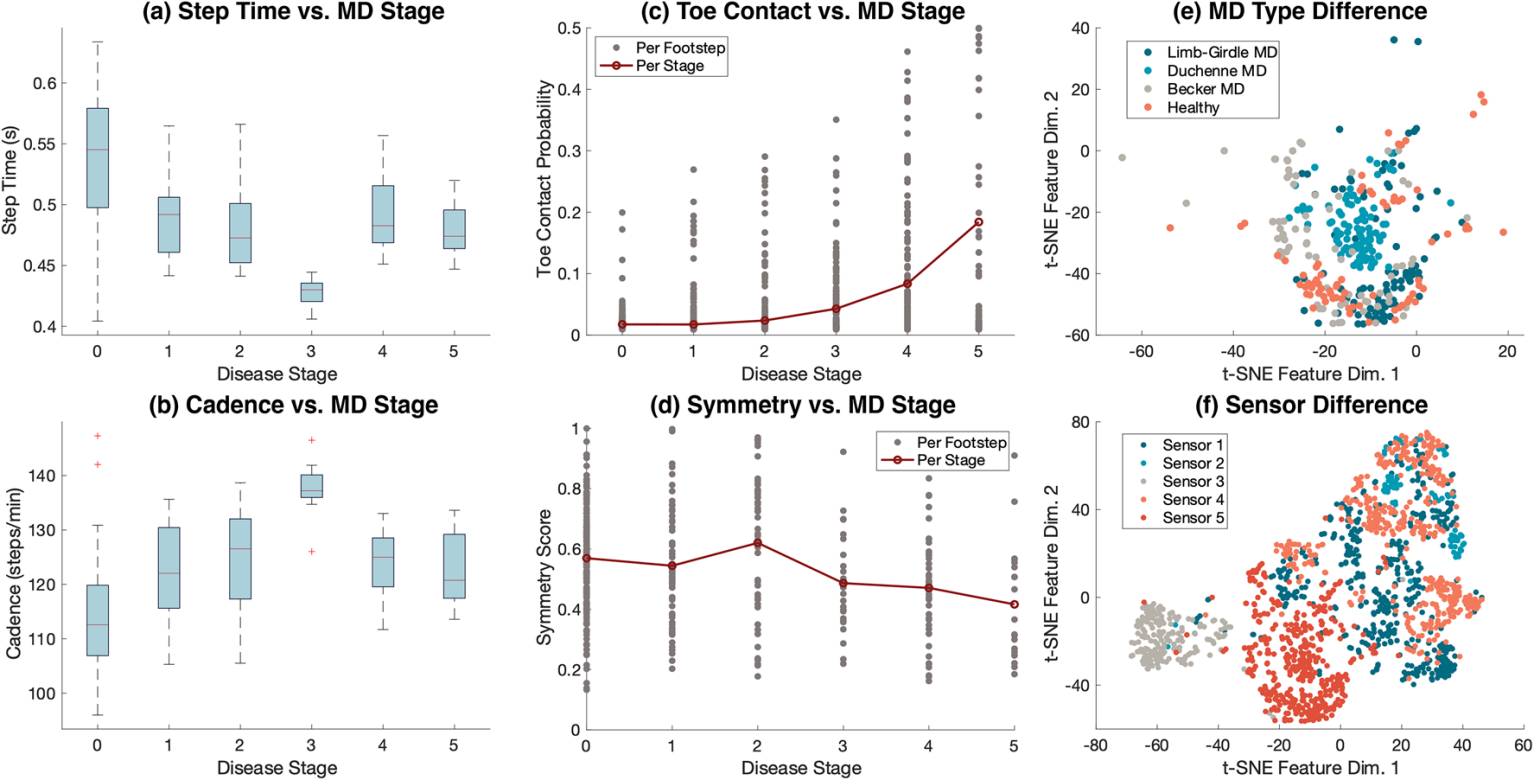
Analysis of symptom-based features relating to MD stages and visualization of MD type and sensor difference in vibration data. (**a**, **b**) The step time and cadence estimated from our data appear to first decrease and then increase as the MD progresses, which may result from the alternation of gait from heel to flatfoot to toe contact along with the weakness in muscles. Note that the data from subjects in stages 0 and 3 are biased by age and individual habits. (**c**) The mean and variance of the toe contact probability increase consistently as the MD stage increases. (**d**) The estimated symmetry score from our data shows less symmetry as the MD stage increases, which may result from weaker muscle control in lower limbers. (**e**) t-SNE plot obtained from self-supervised feature embeddings of gait from various MD types recorded by one sensor. The plot shows distinct gait patterns among MD types compared to healthy gait. (**f**) t-SNE plot obtained from 5 sensors’ data recorded during the second field experiment at the hospital corridor.

Analysis results demonstrate that symptom-based features have consistent trends: as the MD stage increases, (1) step frequency first increases and then decreases, (2) the probability of toe contact increases, and (3) the symmetry score decreases (Fig. 5). In the following paragraphs, we discuss the trends of step time, cadence, probability of toe contact, and symmetry score to show that our approach is interpretable in terms of these gait symptoms.

Existing studies found that as individuals with Duchenne muscular dystrophy moved from the early to late phase, the gait pattern demonstrated decreased walking velocity and cadence, lack of heel contact during the stance phase, and an increased foot drop in the swing that leads to instability^32,33^. The symptom-based features from our study have consistent trends as the above-mentioned results.

As the MD progresses, step time and cadence extracted from floor vibration indicate that the subjects appear to have an increasing trend of step frequency at the beginning and then a decreasing trend as the stage increases, which may be due to the change in initial contact type from heel to toe (Fig. 5a, b). For example, the slight increase in cadence may be caused by the alternation of gait from heel to toe strike which typically has a shorter contact time. As the MD stage increases further, the decrease in cadence may be due to the further degeneration of leg muscles, making the toe-walking much slower^34,35^. However, it is worth noting there is only one subject in stage 3, who has a fast walking habit, which may also lead to a high cadence/short step time and small variance in the fourth bar. In addition, the majority of the healthy participants recorded are adults, who typically have longer step times than children, resulting in longer step times and lower cadence among the stage 0 samples. Therefore, the trends between step time/cadence and MD stages may require more experiments to validate in the future.

The probability of toe contact increases and the symmetry score decreases as the MD stage increases, which are visualized through the scatter plot of individual footstep samples (Fig. 5c and d). The figure shows the estimation value of each sample because the extreme values (samples out of three standard deviations) are also representative of gait health in terms of MD stages. Overall, the estimated mean probability of toe contact significantly increases as the MD stage increases (Fig. 5c). This observation is consistent with previous clinical studies^36,37^, which found that toe-walking is a common symptom in patients with MD, especially after the initial stage. It is worth noting that the number of footsteps with high probabilities also increases, indicating that more footsteps are likely to be toe contacts in patients with MD. In addition, the estimated symmetry score gradually decreases as MD progresses (Fig. 5d), which is also consistent with the previous findings^38,39^. In this case, the number of footsteps with high symmetry scores decreases significantly as the stage increases, meaning that fewer left–right footstep pairs are symmetrical in patients with MD.

### Discussion on the effects of MD types and sensor locations

The vibration data pattern observed from individuals with various MD types aligns with the clinical understanding of the differences among MD types (Fig. 5e). For example, existing studies have found the Limb-Girdle MD typically has a slower expected disease progression compared to Duchenne MD and Becker MD^40^. When plotting the data from individuals of similar ages (12–14 years old), we observe that there is little deviation between the data from the patients with Limb-Girdle MD (dark blue color) and the data from the healthy children (orange color). In contrast, the data from Duchenne MD (light blue color) forms a distinct cluster from the others as it has a significant influence on the shank, which causes more observable changes in gait. While Becker MD (gray color) also affects the shank, it typically has less severe symptoms than Duchenne MD (light blue color), making a proportion of the data closer to the healthy gait (orange color) yet still distinguishable from the other types. Although the study explored various MD stages and types, it is pertinent to note the relatively small number of subjects within the later stages among the Becker and Limb-Girdle MD subgroups. This highlights the potential for further research to offer a more exhaustive exploration of gait-induced floor vibrations within these groups.

In addition, the effect of sensor locations on vibration data can be observed from the data distribution shifts across various sensors (Fig. 5f). The difference across sensors can come from three sources, summarized from a few existing studies^41,42^: (1) floor heterogeneity during vibration wave propagation, (2) location-dependent environmental noises, and (3) hardware variations (e.g., fabrication quality, sensor-floor coupling). In our study, we minimized the environmental noises and hardware variations by inspecting the surroundings and selecting sensors with consistent signals during preliminary tests on pure noise data. However, floor heterogeneity is unavoidable as it is determined by the user’s site. Therefore, we include the sensor number in the step-level features to assist the hierarchical model in recognizing the data distribution shifts across various sensors at the initial level of aggregation. This allows the model to automatically capture the correlation between the sensor and the data during training.

### Discussion on the variability of floors, shoes, and participants

The floor vibration induced by human gait is not only influenced by gait patterns, but also by the floor properties, shoe types, and the participants’ body configurations^43^. For example, existing studies have found that the difference in floor properties results in shifts in vibration frequency ranges due to the changes in stiffness and mass of the floor material^41,44^. The method developed in these studies can be used to generalize the results in our study to more floor types. In addition, our preliminary work on the shoe type effect has found changes in the frequency spectrum of individual footsteps (i.e., step level features), but it does not have a significant effect on the estimation of the walking speed and symmetry in the trace level. Further studies on the shoe effect on initial contact type estimation are needed to enhance the robustness of the method. Moreover, the participant’s body configurations also contribute to the resultant floor vibration, as discussed in previous studies^45^. The body effect (e.g., height, weight) has been considered in this study through the development of person-level feature aggregation in our hierarchical model.

### Broader impacts of ambient floor vibration sensing for gait assessments

The use of footstep-induced floor vibration sensing for gait analysis has potential broad impacts on the surveillance of other gait-related diseases, as well as promoting in-home physical health monitoring. For instance, this technology can be used to assist with understanding the impact of disease progression and other treatments of more common neurodegenerative diseases such as Parkinson’s and Alzheimer’s disease, which affect the gait patterns of individuals at an early stage. By continuously monitoring an individual’s gait patterns, healthcare professionals can detect changes in gait, which indicate the progression of these diseases or the effects of a particular treatment. In addition, the technology can be used for monitoring other conditions that affect gait, such as osteoarthritis and stroke. Such gait information will allow healthcare professionals to assess the severity of gait-related conditions in finer-granularity and tailor treatment plans to the individual’s needs. Furthermore, this method offers a non-intrusive and low-cost approach to collecting gait features, which can be particularly useful for long-term monitoring of individuals in their homes or elder care facilities. By detecting changes in gait patterns, healthcare professionals can also develop interventions to mitigate fall risks and other adverse health outcomes in older adults. Further studies are encouraged to adapt the method developed in this study to in-home environments to address the remaining challenges brought by complex physical environments and diverse individual needs. Overall, the use of ambient floor vibration sensing has the potential for ubiquitous and accessible gait analysis and physical health monitoring, by bringing functional gait assessments to local clinics and individuals’ homes.

## Methods

### Experiment design

The design of the experiment aimed to explore the ability of floor vibration sensing to estimate gait parameters, extract gait symptoms, and track the functional stages of MD. We hypothesized that the floor vibration signals would be able to extract temporal and spatial gait parameters, detect gait symptoms related to MD, and produce reliable predictions of MD stages. Therefore, we designed a 2-phase experiment to test this hypothesis. The first phase was a controlled experiment with healthy subjects in a controlled laboratory setting where the performance was validated with reliable ground truth from the VICON Motion Capture System. The second phase was a real-world experiment with both healthy participants and children with MD, which was conducted in a corridor at a children’s hospital to provide convenience for the participants during their regular clinical visits. All participants have provided written informed consent and/or assent and all experiments are conducted in accordance with the approved protocols under relevant guidelines and regulations of Stanford Institutional Review Board (IRB) and University of Michigan Institutional Review Board (IRB) (with approved protocol number IRB-55372, Single IRB-67992, and HUM00209461) approved the study.

The laboratory experiment was designed to evaluate the feasibility of using floor vibration signals for gait parameter estimation and symptom characterization. Healthy adults (n = 10, 4 male and 6 female; age = 21–40 years; body mass = 47–80 kg; height = 161–180 cm) completed 20 trials of natural walking and 3–10 trials of abnormal walking on a 7-m long platform (Fig. 1a left). The platform in our laboratory is made of laminated wood, which was designed to approximate the typical flooring used at home and was manually constructed by connecting wooden slabs and supporting structures with bolted steel plates. Four geophones were placed on the floor surface along the edge of the walkway with a sampling frequency of 500 Hz. The abnormal walking conditions were completed in the order of toe and midfoot initial contact, asymmetry walking with one leg wearing an ankle brace, and slow walking. The experiment was stopped when the number of trials reached 10 or when the subject requested a rest. Ground truth records were collected with 10 VICON Motion Capture cameras with a sampling frequency of 100 Hz. The geophones were connected with the VICON Locker Lab to enable synchronization^21^. 16 markers were used according to the marker placement specified by the VICON Plug-in Gait lower body model^24^. The VICON data were processed through a built-in pipeline to crop the trials, fill in the missing values, and smooth the moving trajectories of each marker. The foot strike and foot off time were manually marked by an expert based on the reconstructed foot contact and foot off trajectories. The gait parameters were computed through the Plug-in Gait model^24^, serving as the ground truth for evaluation.

The field experiments include two sensor layouts (Fig. 1a right). In the first experiment layout, patients with MD (n = 7, 4 male and 3 female; age = 7–15 years; body mass = 16–54 kg; height = 102–163 cm) and healthy subjects (n = 6, 4 male and 2 female; age = 7–15 years; body mass = 41–85 kg; height = 143–183 cm) each completed 5–10 natural gait trials per person along a 15-m hospital corridor. In the second experiment layout, patients with MD (n = 8, 7 male and 1 female; age = 7–15 years; body mass = 17–51 kg; height = 106–161 cm) and healthy subjects (n = 5, 3 male and 2 female; age = 13–27 years; body mass = 35–88 kg; height = 125–182 cm) each completed 5–10 natural trials per person along a 10-m hospital corridor. The hospital corridor is made of concrete with vinyl tiles on the surface, which is one of the most popular floor types for indoor living spaces. For both experiments, each trial was stopped when the number of trials reached 10 or when the subject requested a rest. 5–8 geophones were placed on the floor surface along the edge of the walkway with a sampling frequency of 25600 Hz to maximize the temporal resolution. 3 and 5 video cameras were mounted at various locations along the corridor (Fig. 1a), aiming at the lower body of the walking subjects to collect the ground truth of gait symptoms. A hammer strike was conducted at the beginning of each trial to enable synchronization of the video and vibration data. The video data were processed through manual cropping, MD symptom observation by an MD specialist, and footstep counting for each trial. Ground truth of functional assessments on MD progression was obtained by medical practitioners from the children’s hospital, which include the time to run 100 m, North Star Ambulatory Assessment (NSAA), and North Star Assessment for limb-girdle type muscular Dystrophy (NSAD)^46^.

### Development of a unified functional scale for patients with MD

A unified scale for MD progression was defined based on the 100-m run time measured during the functional assessments conducted in clinics. Running speed is a simple, well-established means to grossly quantify ambulation abilities^47,48^. The 100-m timed tests were recently introduced for use in individuals with neuromuscular disorders such as DMD and LGMD as it is a simple-to-understand concrete disease that can be used in adults and children^20,49^. A unified scale to monitor their progression is needed because the purpose of developing the floor vibration sensing approach is to advance the accessibility of gait assessments for general patients with various MD types. The scale was defined by dividing the performance based on the normative time of a 100-m run defined in the previous study^20^. The normative 100-m run time was computed by comparing the actual time with the 50th percentile time among the healthy controls, normalized by age, height, and weight:

1 $$\begin{aligned} \quad Predicted \quad 100m\% = \frac{36.72 - 1.51 \times age (year)+ 0.26 \times \frac{weight (kg)}{height (m^2)}}{Actual\quad 100m\quad Time} \times 100\% \end{aligned}$$

where the predicted 100m % is the normative value ranges from 0% to 100%. The numerator is a running speed prediction mode developed through a regression analysis^20^. A 100% value means an individual’s running time is the mean of the corresponding age group. The continuous value of predicted 100m % was further discretized into several divisions, forming a reference of MD progression stage ranges from 0 to 7:

**Table:** 

MD stage	Predicted 100 m%	Interpretation
Stage 0	100	Faster than 50th percentile of the control subjects.
Stage 1	80–99	Slower than 50th percentile of the control subjects. Could be early signs of functional decline.
Stage 2	60–79	Prone to walk slightly slower and may have flatfoot/toe contact.
Stage 3	50–59	Prone to walk slower. Likely to have gait abnormalities such as shorter stride length and a wider base of support.
Stage 4	40–49	May have noticeably adapted gait patterns and may use wheelchairs part of the time.
Stage 5	30–39	Likely to use wheelchairs for part of the day.
Stage 6	20–29	Difficulty walking outside of their homes. About to lose walking ability in several years.
Stage 7	0–19	Likely to lose walking ability.

### Vibration data processing

The floor vibration data were processed through an algorithmic pipeline where footstep-induced signal impulses were detected and filtered. To detect the footstep-induced impulses in the ambient floor vibration, a peak detection algorithm was developed, which takes a section of pure noise signal as a reference and applies a 0.1-s sliding window over the streaming data to identify the peaks in the vibration signals by comparing the data with that of the pure noise. Impulse peaks with a mean amplitude more than three standard deviations above the noise mean were detected. Then, the duration between the impulses and the number of consecutive impulses were extracted to determine whether they were induced by footsteps or not. Since footstep-induced signal impulses have a typical duration between 0.2 and 0.6 s, longer or shorter signals were first excluded. Unlike impulses induced by door closing or item dropping, footstep-induced signals typically have more than three consecutive peaks as the person passes by. Therefore, signals with less than three consecutive impulses were also excluded from the dataset.

After detecting and isolating the footstep-induced vibration signals from the ambient floor vibration, the signals were then filtered by a lowpass filter and Wiener filter to reduce noises. The lowpass filter aims to reduce the high-frequency sensory noises and exclude the information that is not relevant to gait. A 500 Hz threshold was chosen because 97% of the signal power was included in the 0–250 Hz frequency range. The Wiener filter was used to reduce the environmental noises by subtracting the spectrum of pure noise from that of the footstep-induced vibration signal. The processed vibration signals were stored on an encrypted cloud drive where each sample describes an individual trace that consists of a series of consecutive footsteps from a person, recorded by one sensor.

Before feature extraction and data modeling, the samples were processed through a re-sampling algorithm to balance the sample number among various subjects for better generalization. This resampling algorithm integrates bootstrapping and down-sampling, where the median sample size serves as a benchmark for adjusting the sample sizes of other subjects accordingly. This strategic adjustment improves the sample efficiency while reducing redundancy within the dataset, aiming to improve the model performance.

### Physics-informed gait pattern extraction from floor vibration signals

Floor vibrations generated by footstep forces contain rich information about the gait and the floor. Similar to hammer strikes, each footstep can be regarded as a short-duration force applied to the floor, causing a small deformation in the underlying floor slab. While the displacement is difficult to observe by the human eyes, it changes the internal stress distribution of the slabs, resulting in dynamic floor movements to retain equilibrium. As the ensuing vibration response waves propagate through the floor, they can be measured by vibration sensors attached to the floor surface similar to how seismologists measure earthquakes. The geophone sensors transform the vertical displacements of the floor into electrical voltage time series. If there is a slight change in the footstep force or foot-floor contact surface due to gait symptoms related to MD, the floor vibration response changes accordingly, as described in an existing study using floor vibration sensing^13^. Therefore, we leverage such changes to infer physical characteristics of gait patterns from individuals with MD.

**Step time and cadence estimation.** The method to extract step time and cadence from floor vibrations leverages the empirical observation that each footstep induces only one vibration impulse at the time when the foot strikes the floor. Therefore, we extract the start time of the impulse to represent the foot strike time and count the number of impulse peaks within each trace to estimate the cadence. The specific process involves four steps. First, we apply a wavelet transform using the Morse wavelet (an efficient and commonly used wavelet type) to obtain the time-frequency relationship in the vibration signals. Then, we isolate the frequency range where the natural frequency of the floor lies (typically 5–25 Hz^41,44^) to filter out noises caused by mechanical device disturbances from the environment. The wavelet coefficient series is then processed through an anomaly detection and peak-picking algorithm to extract the foot strike time and the number of footsteps. Finally, we compute the step time by subtracting the foot strike time between adjacent steps, and the cadence through a division of the number of footsteps by the duration of the trace.

2 $$\begin{aligned} Step\quad Time_i= & {} t_{footstrike}^{i+1} - t_{footstrike}^{i}, \forall i=1,2,...,N \end{aligned}$$

3 $$\begin{aligned} Cadence= & {} \frac{N-1}{t_{footstrike}^{N} - t_{footstrike}^{1}} \end{aligned}$$

where *N* is the number of footsteps in a recorded trace.

**Symmetry score estimation.** The approach to extract the symmetry score is developed based on the insight that a larger footstep force typically induces a higher amplitude in floor vibration at the same location. Since the two adjacent footsteps have relatively closer locations than the other footsteps along the walking path, we assume that the wave attenuation effect is less dominant than the effect of the footstep force differences for the two adjacent steps. Therefore, we extract the symmetry score based on the 90th percentile of the signal amplitude of each footstep and take the mean score among the trace to reduce the wave attenuation effect for different left–right pairs. The 90th percentile instead of the maximum value to minimize the estimation bias caused by signal clipping or extreme values due to noises. The symmetry score is estimated as follows:

4 $$\begin{aligned} Symmetry \quad Score = 1 - \frac{1}{N-1} \sum _{i=1}^{N-1} \frac{|X_{90}^{i+1} - X_{90}^{i}|}{0.5(X_{90}^{i+1} + X_{90}^{i})} \end{aligned}$$

where $X_{90}^{i}$ is the 90th percentile of the signal amplitude for $i^{th}$ footstep and *N* is the number of footsteps in a trace. The higher the value is, the more symmetrical a person’s gait is.

**Initial contact prediction.** To examine how various initial contacts affect the resultant floor vibration, we formulate the dynamical system of foot-floor contact to extract features that are representative of initial contact types. We assume that each footstep exerts a force $F_l(t)$ at a simply supported beam within the linear elastic range to simplify the complex situation^50^. The equation of motion suggests:

5 $$\begin{aligned} M\ddot{u}(t) + C{\dot{u}}(t) + Ku(t) = F_l(t) = -S(t)\ddot{u}_f(t) \end{aligned}$$

where *M*, *C*, *K* are the mass, damping, and stiffness matrix of the floor. $F_l$ is the footstep force, which can be further expressed by the equivalent spatial loading matrix *S*(*t*) and the foot acceleration $\ddot{u}_f(t)$. In order to simplify the complex temporal and spatial dependency in the above equation, we focus on the instant time when the initial contact happens and assume independence between temporal and spatial variables (i.e., $S(t) = S$).

The above derivation suggests that the floor vibration signal is determined by the spatial distribution of the footstep force and the amplitude and direction during the contact. This formulation validates the hypothesis from a theoretical perspective that we can still infer the characteristics of initial contacts from the frequency components of floor vibration signals, without knowing the exact properties of the floor.

Therefore, we infer the probability of toe contact by training a data-driven model based on the frequency spectrum of the vibration signals from each footstep. The data-driven model leverages the support vector machine with a quadratic kernel to capture the inter-dependency between various frequency ranges. The output of the data-driven model is the type of contact. In our model, we translate the confidence score of the prediction into the probability of toe contact as the initial contact feature.

### Hierarchical feature modeling through multi-layer neural networks

The developed hierarchical model involves three multi-layer neural networks to aggregate the features. Since the feature dimension and sample size decrease as they are aggregated through the hierarchy, the scale of the model also decreases. This is because a balanced ratio between the sample number and feature dimensions enables efficient learning and can effectively mitigate problems such as over-fitting. In this study, the first neural network has three layers, each with 12 (input dimension), and 256, 64 neurons. The second neural network has three layers, with 20 (input dimension), 128, and 32 neurons. The third neural network has two layers with 9 (input dimension) and 64 neurons. The third neural network can also be replaced by a simpler model architecture, such as k-nearest neighbor classifiers, decision trees, and support vector machine classifiers because the feature embeddings are already distinguishable among different classes as observed in Figure 4c. The operations in $l^{th}$ layer includes a linear transformation $h_l$ and an activation $a_l$:

6 $$\begin{aligned} h_l= & {} w^T_l x + b_l \end{aligned}$$

7 $$\begin{aligned} a_l= & {} ReLu(h_l) = max(0, h_l) \end{aligned}$$

where $w_l, b_l$ are model parameters. We choose the commonly used “ReLu” activation because it introduces non-linearity to a neural network and solves the vanishing gradients issue, which has been shown to enable the model to learn faster and perform better^51^. The output from the last layer in each hierarchy is then aggregated by computing the mean value and then concatenated with the features in the next hierarchy. For example, all footsteps in the same trace were aggregated by computing the mean among all of them and then concatenated with the trace-level features. Specifically, if the $j^{th}$ trace has a set of footsteps $T_j$, then input to the trace-level neural network is the concatenation of trace-level features and step-level aggregations:

8 $$\begin{aligned} h_j = concat(h_j^{trace},h_j^{steps}) \end{aligned}$$

where

9 $$\begin{aligned} h_j^{steps} = Mean(h_i, i \in T_j) \end{aligned}$$

The hierarchical neural network is trained hierarchically in two steps. First, the neural network at each hierarchy is pre-trained with labels before aggregation with a cross-entropy loss commonly used for classification tasks. This allows “teacher enforcing”, which means each neural network can effectively learn the features related to MD progression. Compared to the architecture where the labels are only applied at the final hierarchy, the “teacher enforcing” step enables more efficient learning with less variance and error propagation. After that, the entire model is trained together to allow information sharing among multiple hierarchies. A softmax function^52^ was used to normalize the embedding from the previous hierarchy before it was concatenated with the normalized features in the next hierarchy. In addition, a 20% dropout was applied to the model to mitigate overfitting, where the rate is determined through empirical tests among various dropout percentages to optimize the test performance. The final output is a vector representing the probability of a person’s footsteps belonging to each progression stage. The progression stage with the highest probability was determined as the final prediction.

### Evaluation metrics

The evaluation metrics in this study are the mean absolute error for estimating continuous values of gait parameters, and the F-1 score to evaluate and compare the performance of classification.

**F-1 Score.** The F1 score is a measure of a model’s accuracy on a dataset. It is commonly used to evaluate classification systems when the samples in each class are imbalanced. Since the MD stages among our test subjects are imbalanced, the F1 score is chosen to balance between false negatives and false positives^53^. The F1 score is the harmonic mean of precision and recall, and thus symmetrically represents both in one metric. The precision and recall are computed based on the number of true positives (TP), false positives (FP), and false negatives (FN) for each class. For a multi-class classification problem in our study, we calculate the average of F-1 scores among all classes. For each class, the F-1 score is computed as follows:

10 $$\begin{aligned} F-1 \quad Score = \frac{2\times Precision \times Recall}{Precision + Recall} \end{aligned}$$

where

11 $$\begin{aligned} Precision = \frac{TP}{TP + FP}, \quad Recall = \frac{TP}{TP + FN} \end{aligned}$$

The F-1 score ranges from 0 to 1. A higher F-1 score typically means better prediction accuracy. In this study, the mean F-1 score is used to evaluate the performance of initial contact and symmetry prediction, as well as the estimation of MD stages.

**Mean absolute error.** The mean absolute error (MAE) is a frequently used measure of the differences between values predicted by a model and the ground truth values. Given *N* observations, let the prediction and ground truth for each data point be $\hat{x_i}$ and $x_i$, the MAE is defined as follows:

12 $$\begin{aligned} MAE = \frac{\sum _{i=1}^{N} |x_i - \hat{x_i}|}{N} \end{aligned}$$

MAE is the standard deviation of the residuals, which describes how far the predicted value is from the true data points. MAE is a direct measure of the mean of these residuals: a smaller value means a more consistent match between the prediction and the ground truth. Since MAE has the same unit as the original variable, it provides an intuitive comparison between the prediction and the ground truth, which is more effective in capturing the overall model performance than the root-mean-square error (RMSE) metric^54^. Therefore, we choose MAE to estimate the accuracy of step time and cadence estimation.

## Data Availability

Sample datasets of the healthy subjects from all three studies are made publicly available at https://zenodo.org/record/8125744. The use of patients’ data and personal biometric information in further scientific work will require a data-sharing agreement with the hospital.

## Code availability

Data processing and feature extraction were performed using custom Matlab scripts. The model was implemented as custom scripts in both Python and Matlab. All codes are made publicly available along with the dataset at https://zenodo.org/record/8125744.
